# The cortical energy needed for conscious perception

**DOI:** 10.1016/j.neuroimage.2008.01.032

**Published:** 2008-05-01

**Authors:** Marieke L. Schölvinck, Clare Howarth, David Attwell

**Affiliations:** Department of Physiology, University College London, Gower Street, London, WC1E 6BT, England

## Abstract

The brain's information processing power is limited by its energy supply but the allocation of cortical energy use between conscious and unconscious information processing is unknown. We calculate, from electrophysiological data in primates, that conscious perception reflects surprisingly small local alterations in mean cortical neuronal firing rate and energy consumption: perceiving visual stimulus movement, altered tactile vibration frequency, or tone stream separation, changes local cortical energy use by less than 6%. Our estimations of energy use suggest that a “design strategy”, of encoding signals using separate neurons that increase and decrease their firing rate, serves to minimise changes of energy use in the cortical areas mediating perception and may result in stimulus perception failing to be detected by BOLD functional imaging.

## Introduction

Neuronal activity is highly energy demanding, and energy supply is a major constraint on the information processing power of the brain (Attwell and Laughlin, 2001). Accordingly, to distribute glucose and oxygen to the brain areas that need them most, blood flow is increased in regions where neurons are active. The need for this increased blood flow is suggested by traditional analyses of sensory systems, which assume that external stimuli evoke action potentials in neurons that have receptive fields “tuned” to particular features of the stimuli (Hubel and Wiesel, 1998): on this basis, low levels of spontaneous neuronal activity merely provide a “noise” background against which the signal must be detected (Barlow, 1957), and sensory input should greatly increase cortical energy use. Recently, however, it has been suggested that incoming sensory information produces only small changes to the ongoing dynamic pattern of activity in cortical neurons (Arieli et al., 1996, Fiser et al., 2004), implying that sensory input alters cortical energy use only slightly.

The blood flow increase associated with increased neuronal energy use is the basis of functional imaging techniques such as positron emission tomography (PET) and blood oxygen level dependent functional magnetic resonance imaging (BOLD fMRI). However, blood flow measurements cannot be used to calculate the energy usage supporting a cognitive task, because the blood flow increase is generated by a variety of neurotransmitter-mediated mechanisms (Drake and Iadecola, 2007) rather than being driven directly by energy use. In brief, glutamate released by neural activity activates a Ca^2+^ flux into neurons which activates nitric oxide synthase leading to release of the vasodilator NO (Faraci and Breese, 1993), and also activates metabotropic glutamate receptors on astrocytes which leads to arachidonic acid production by phospholipase A_2_ and thus to the release of vasodilatory prostaglandins (Zonta et al., 2003). Furthermore, there may be complex changes in the fraction of energy provided by glycolysis and oxidative phosphorylation during and after neural activity (Fox et al., 1988, Gjedde et al., 2002; Kasischke et al., 2004). In addition to this, the blood flow response following neural activation occurs over a volume significantly larger than that of the activated neural tissue (Malonek and Grinvald, 1996).

**Fig. 1:**
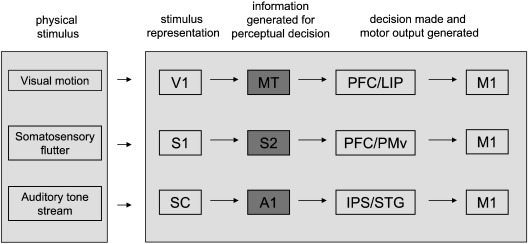
Brain regions involved in the perceptual tasks considered in this paper. Incoming stimuli are represented as neural firing in areas such as V1, S1 and SC. Areas MT, S2 and A1, which we analyse here, are the first areas where firing rate provides information allowing a decision on the presence of a percept of visual movement, altered skin vibration (“flutter”) frequency or the grouping of sounds (“tone stream segregation”). Subsequent areas may make the perceptual decision and generate motor output (note that although we show a pure feed-forward flow of information, in reality there are also reverse interactions between different areas which probably contribute to the perceptual decision). V1, primary visual cortex; S1, primary somatosensory cortex; SC, superior colliculus; MT, middle temporal area; S2, secondary somatosensory cortex; A1, primary auditory cortex; PFC, prefrontal cortex; LIP, lateral intraparietal area; PMv, ventral premotor area; IPS, intraparietal sulcus; STG, superior temporal gyrus; M1, primary motor cortex.

We therefore estimated, from electrophysiological data in primates, the local cortical energy use associated with conscious perception of a stimulus property in three different sensory modalities, vision, touch and hearing (Fig. 1). We adopted a philosophy similar to that in many functional imaging experiments, by subtracting the energy use powering neural responses to two types of stimuli which differ only in the conscious perception of a particular attribute of the stimulus (Tootell et al., 1995; Phillips et al., 1997; Epstein and Kanwisher, 1998; McKeefry and Zeki, 1997). Because of the importance of conscious perception for determining behaviour, one might expect that the perception of a stimulus attribute would be associated with a set of neurons increasing their firing robustly. However our results show that, although the unconscious representation of information from which a percept is derived can involve large changes of neural firing rate, conscious perception, i.e. the change from not being able to distinguish two stimuli to perceiving them as different, is associated with a surprisingly small change of mean neuronal firing rate and energy consumption.

In what follows, the detailed calculations of firing rate changes, and justification of the assumptions made, are presented in Materials and methods (which can be omitted on a first reading), with a full description of the perceptual tasks considered and the resulting conclusions about changes in cortical energy use being presented in Results.

## Materials and methods

### Overview of approach

We identified tasks that have been studied psychophysically and electrophysiologically in monkeys, as well as psychophysically and by functional imaging in humans. These tasks were detection of movement of random dot stimuli, detection of flutter vibration applied to the skin, and detection of auditory tone stream segregation, and are described in detail in Results. Electrophysiological single unit data from monkeys were used to evaluate the change in mean neuronal firing rate occurring in cortical areas providing neural responses which can be used for the detection of visual motion, skin vibration frequency or sound frequency (Fig. 1). This change in firing rate was then converted into an estimated fractional change of energy use in that area, using an energy budget for the cerebral cortex (Attwell and Laughlin, 2001) which predicts the energy consumed on different subcellular processes underlying neural information processing. For each task (and associated brain area) considered the aim was to evaluate the change in action potential firing, and hence the extra energy needed, for perception, above that needed merely to represent stimuli which do not produce a percept, since this is the change in energy consumption between brain activation states that are commonly compared in functional imaging experiments (Tootell et al., 1995; Phillips et al., 1997; Epstein and Kanwisher, 1998; McKeefry and Zeki, 1997).

To convert the altered firing rate to a change of energy usage, we employed the fact that the increase in energy used for signalling in the cells recorded from, and in the input neurons driving their firing, can be divided into two parts, proportional to postsynaptic and presynaptic action potential frequency respectively (Attwell and Laughlin, 2001). Energy used to reverse the ion movements generating postsynaptic action potentials is proportional to postsynaptic firing rate (Attwell and Laughlin, 2001). Energy used on presynaptic action potentials and transmitter release, transmitter and vesicle recycling, and reversal of postsynaptic ion movements generated by transmitter release, is proportional to presynaptic firing rate (Attwell and Laughlin, 2001). For simplicity, in what follows we assume that the input–output relationship of the synapses impinging on the cells recorded from is linear, so that the fractional change of firing frequency in the recorded cells is equal to the fractional change of presynaptic frequency in their input neurons (this assumption is assessed below). This implies that the fractional change in total firing-related energy use is equal to the fractional change in the firing rate of the recorded postsynaptic cells. However, in addition to these action potential driven processes, 25–50% of brain energy is also expended on the resting potentials of neurons and glia, and on non-signalling housekeeping tasks (Attwell and Laughlin, 2001).

In the first three sections below, we assess the change in firing rate of cortical pyramidal cells when a stimulus is at the threshold of conscious perception for the perceptual tasks considered in the three cortical areas. This is followed by an explanation of how we converted changes in pyramidal cell firing rate into changes of cortical energy use, and an assessment of the change in firing rate and energy use for perception of reliably detected stimuli (i.e. stimuli that are well above threshold).

### Detection of visual motion

**Fig. 2:**
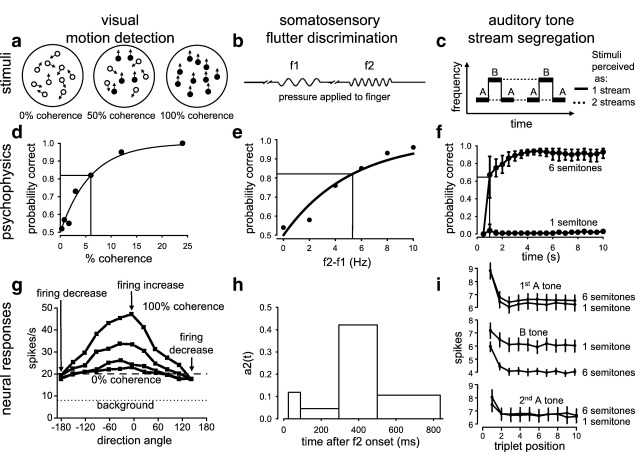
Perceptual tasks (a–c), psychophysical responses (d–f) and cellular responses (g–i) analysed in the text. (a–c) Stimulus schemata for the three tasks considered (adapted from Rees et al. (2000), Romo et al. (2002), Micheyl et al. (2005)). (a) Three different levels of coherence in the visual motion task. At 0% coherence, all dots move randomly. As coherence increases, more dots move in a coherent fashion in one direction (black symbols) while the others move randomly. (b) Somatosensory task, showing a frequency discrimination trial where the second stimulus (*f*2) has a higher frequency than the first stimulus (*f*1). (c) Auditory percepts during stream segregation. The stimuli (repeating triplets of different frequency tones, ABA) are perceived as a single stream of connected tones (solid lines) or as two monotonic streams with different tempi playing in parallel (dashed lines). (d–f) Psychometric curves for visual motion (d), somatosensory (e), and auditory (f) tasks (from Britten et al. (1992), Romo et al. (2002) & data in Micheyl et al. (2005) provided by C. Micheyl respectively; curves in d and e are fits of Eq. (1) of Materials and methods). Black lines indicate threshold values for perception, for the random dot coherence, difference in flutter frequency (*f*2–*f*1), and time for build-up of auditory stream segregation (but in d and e the exemplar thresholds differ from the mean values used in the calculations). (g–i) Neuronal responses evoked by the tasks (adapted from Britten and Newsome (1998), Romo et al. (2002), Micheyl et al. (2005)). (g) Direction tuning functions for an MT neuron at motion coherences of 12.8, 25.6, 51.2, and 100% (bottom to top; frequency scale adjusted so that background firing rate (8 Hz) and rate with a 0% coherence stimulus (20 Hz) are those found on average: see text). For stimuli close to the preferred direction, increased coherence increases firing, but as the orientation approaches 180^o^ to the preferred direction, the rate decreases below the rate with a 0% coherence signal (arrows). (h) Average firing rate parameter a2 (see Materials and methods) for S2 neurons, derived by fitting the Eq. firing rate = *a*1^⁎^*f*1 + *a*2^⁎^*f*2 + constant, during *f*2 presentation. The dependence of the firing rate on *f*2 changes over time. ( i ) Spike counts (per 125 ms) evoked in A1 neurons with best frequency A, by the A and B tones in repeating ABA triplets.

To calculate the energy needed to support the perception of motion in area MT (Figs. 1, 2a; see Results for a full description of the task), we need to know the action potential rate of the cells analysing the motion. Monkey MT neurons have a firing rate of ~ 8 Hz in darkness, and this increases to 20 Hz when the animal views a 0% coherence stimulus of moving dots (analysis (Huk and Shadlen, 2005) of data in Britten et al. (1993)). Increasing the coherence of the stimulus increases the firing of cells tuned to respond to the direction of motion of the coherently moving dots, and decreases the firing of cells tuned to the opposite direction (Fig. 2g). Psychometric functions (Fig. 2d) measured psychophysically, or “neurometric” functions derived by deducing the psychophysical performance of an ideal observer comparing the responses of neurons tuned to the direction of motion and to the opposite direction, can be fitted by the function

1 $$p=1-0.5\;\exp\;(-{\left(c/\alpha \right)}^{\beta })$$

where *p* is the probability of correctly detecting the motion direction (out of 2 oppositely directed possibilities) at coherence *c*, *α* is termed the threshold for detection, and *β* is a measure of the steepness of the function (Britten et al., 1993, Britten et al., 1992). Detection at threshold (*c* = *α*) corresponds to correctly detecting the direction of motion on 1–0.5e^–1^ = 82% of trials (compared to the 50% obtained by chance for a zero coherence signal: Fig. 2d). Experimentally, this 82% success rate for detecting motion can be achieved by comparing the responses of neurons tuned to the direction of motion and of neurons tuned to the opposite direction when stimuli have a coherence of 14% (from the neuron data in Fig. 9A of Britten et al. (1992), weighted by number of cells). Having derived this threshold coherence allows us to calculate the mean firing rate of MT cells when detecting motion.

The action potential rate of MT neurons responding to motion in their preferred direction increases with coherence of the stimulus at a rate of 0.39 Hz/(% coherence), while the rate in cells whose preferred direction is opposite to the stimulus decreases at 0.11 Hz/(% coherence) (Britten et al., 1993). Thus, for 14% coherence, these cells will increase and decrease their rate by 5.47 Hz and 1.55 Hz respectively. Adding the 20 Hz firing occurring in the presence of a 0% coherence stimulus (see above), these cells will therefore fire at 25.47 and 18.45 Hz respectively. A column of MT cells analysing motion in a retinal area (Albright et al., 1984) contains cells responding to all possible directions of motion which, in general, will respond at a rate between these extremes (Fig. 2g). The firing rate can be expressed as

2 $$D\;\exp(-{\left(x/\sigma \right)}^{2})+E$$

where x is the angle relative to the preferred direction, *σ* is the bandwidth of the response and *D* and *E* are constants (Britten and Newsome, 1998) (Fig. 2g). Using *σ* = 88^o^ for 14% coherence (from Fig. 3 of Britten and Newsome (1998)), and fitting this equation to firing rates of 25.47 and 18.45 Hz at 0^0^ and 180^0^, gives *D* = 7.13 Hz and *E* = 18.34 Hz. The mean firing rate averaged over all of the cells in the column is thus

*∫*_0_^180^ \{*D* exp(−(*x*/*σ*)^2^) + *E*\} d*x*/180 = (*D*/2)·(*σ*/180)·(√*π*)·erf(180/*σ*) + *E* = 21.4 Hz}

when the motion is perceived at threshold. In contrast, when viewing a 0% coherence stimulus, the firing rate is 20 Hz (see above). Thus, the motion percept is associated with a 7% increase in mean firing rate {100% × (21.4–20) / 20}.

Perception of motion by MT neurons evokes an increase in firing rate and energy use only because of an asymmetry in the neural responses to motions in the preferred and non-preferred directions of the cells: stimuli in the preferred direction evoke a larger increase in firing than the decrease of firing evoked by stimuli in the opposite direction (Britten et al., 1993). In principle the system need not be constructed with this asymmetry, and it might be possible for perception to occur with no change of mean firing rate and energy usage at all relative to viewing a 0% coherence stimulus (or even a decrease in mean firing rate and energy usage if the relative strengths of the responses in the preferred and opposite directions were reversed).

### Detection of skin vibration

Neurons in monkey S2 (Fig. 1) change their firing when a flutter stimulus is applied (see Results for a full description of the task), but only 40% of these show a response that depends on the vibration frequency (Romo et al., 2002) (of which 57% show an increase in firing rate with increasing vibration frequency, and 43% show a decrease in rate with increasing frequency (Romo et al., 2003)). When two stimuli of different frequency, *f*1 and *f*2, are applied for 0.5 s, separated by a few seconds in time (Fig. 2b), the action potential firing rate response to the second stimulus can be represented as a function of *f*1 and *f*2 as follows (Romo et al., 2002)

3 rate(f1,f2)=a1·f1+a2·f2+constant

where a1 and a2 are experimentally determined functions of time that evolve so that for much of the response a1(*t*) = –a2(*t*) and the rate depends solely on the difference in frequency, *f*2–*f*1. For an initial frequency of *f*1 =  20 Hz, we evaluated the mean difference in spike count (∫rate(*f*1, *f*2) d*t*) averaged over all cells when *f*2 was sufficiently above *f*1 to be distinguished perceptually from a second presentation of a stimulus with *f*2 = *f*1 = 20 Hz. Using the definition of threshold in Eq. (1), i.e. 82% of behavioural choices correct (Fig. 2e), and taking correlations between neuronal responses into account (Romo et al., 2003), the threshold for perceiving that *f*2 > *f*1 is reached when *f*2 is raised from 20 to 24.6 Hz (from Fig. 4 of Romo et al. (2003)), and the resulting increase of spike count from Eq. (3) is

∫rate(f1=20,f2=24.6)ⅆt−∫rate(f1=20,f2=20)ⅆt=4.6Hz·∫a2(t)ⅆt

The motor response defining the monkey's choice of whether *f*2 > *f*1 starts, on average, 836 ms after the start of the *f*2 stimulus (Romo et al., 2002). Mean values of a2(t) were obtained for times 25–90 ms, 90–295 ms, 295–500 ms and 500–836 ms after the start of the *f*2 stimulus by averaging over the experimental data for cells in Figs. 6a, 6b, 6c and 6d, respectively, of Romo et al. (2002), taking into account the fact that 95, 61, 17 and 60 cells, respectively, of the 208 cells studied are not plotted in those figures because a2 was not significantly different from zero (Romo et al., 2002). This procedure gave mean values for a2 of 0.119, 0.046, 0.421 and 0.106 (respectively) for the four time periods (Fig. 2h), and thus a mean increase in spike count of 0.64 action potentials per neuron. This mean increase is very low because similar numbers of cells show increases (for which a2 is positive) and decreases (for which a2 is negative) in firing rate in response to the flutter stimuli (Romo et al., 2002). Since only 40% of neurons show responses dependent on the stimulus frequency (Romo et al., 2002), averaged over all the cells in S2 the mean increase of action potentials/neuron is 0.26, for the 3-s period during which the two stimuli are applied. The baseline firing rate of these cells is ~ 15 Hz (Fig. 7b of Romo et al. (2002), data for *t* < 0), so this corresponds to a 0.57% increase of firing over the 3-s trial.

### Detection of auditory tone stream segregation

The change in energy expenditure associated with tone stream segregation in A1 (Figs. 1, 2c; see Results for a full description of the task) can be estimated as follows. Psychophysical experiments (Micheyl et al., 2005) on the response of humans to ABA triplet tone sequences applied at 0.5-s intervals show that if B is 6 semitones (√2-fold) above A in frequency then stream segregation occurs over a period of seconds, and after 2 presentations of the triplet tone the probability of correctly perceiving the segregated streams reaches 64% (which, since the probability rises from 0 to ~ 100%, is equivalent to the 82% criterion used in Eq. (1) for an experiment where the probability rises from 50 to 100%, as 82% = 50% + (100–50)% × 64%). In contrast, if B is only 1 semitone (6%) above A in frequency, or the same as A, then stream segregation does not occur even after 10 s of the stimulus (Fig. 2f, from Fig. 1 of Micheyl et al. (2005)). A possible neural basis for the phenomenon of tone stream segregation is suggested by experiments on primary auditory cortical (A1) neurons in monkeys (Micheyl et al., 2005; Fishman et al., 2001). For the ABA-ABA-ABA- stimulus, if A and B are sufficiently different, neurons with a best frequency equal to that of the A tone show a different time course of adaptation to the repeated presentation of the A and B tones: although the responses to both tones decrease over the first few presentations of the tone triplet, the response to the off-best frequency B tone is not only smaller but shows more (percentage) adaptation with repeated presentation of the triplet tones (Micheyl et al., 2005) (Fig. 2i). Similarly, for a neuron with a best frequency equal to that of the B tone, the response to the A tones would decrease more over time than the response to the B tone (Micheyl et al., 2005).

Thus, if the A and B tones are sufficiently separated, after some duration of exposure to the tone sequence, high signal to noise ratio responses will only be produced for the A tones in neurons tuned to the A frequency, and for the B tones in neurons tuned to the B frequency. In contrast, when A and B are close, the extra adaptation to the B tone is not seen in neurons tuned to the A frequency (Fig. 2i), and vice versa, so that information on both tones will be encoded with a high signal to noise ratio by both sets of neurons. This representation of the tone sequence in 2 discrete sets of cells when A and B are sufficiently different, but in the same cells when A and B are close, is postulated to underlie the different perceptions produced (Micheyl et al., 2005, Fishman et al., 2001) (Fig. 2c). Human magneto-encephalographic (Gutschalk et al., 2005) and fMRI (Cusack, 2005) studies suggest that auditory areas outside A1 (Fig. 1) may also contribute to the perceptions produced, perhaps by controlling attention to the information streams produced in cells at different locations in A1.

In primary auditory cortical neurons of monkeys with a best frequency of A, Fig. 3 of Micheyl et al. (2005) shows that, when B is 6 semitones above A, the first 2 presentations of the triplet tone sequence evoke 8.82 and 6.79 action potentials respectively in response to the two first A tones (a total of 15.61 spikes), 5.97 and 4.46 action potentials in response to the two B tones (a total of 10.43 spikes, much less than for the A tone because of the adaptation described above), and 7.48 and 6.77 action potentials in response to the last A tones (a total of 14.25 spikes), giving a grand total of 40.29 spikes in response to the 2 triplet sequences (this includes the baseline firing rate of ~ 25 Hz). In contrast, when B is only 1 semitone above A, although the total spikes in response to the two first and two last A tones of the 4 triplets were little changed (15.28 and 14.81 respectively), the total spike number in response to the intervening B tones was increased from 10.43 to 13.69 (because of the absence of the adaptation described above: Fig. 2i), giving a grand total of 43.78 spikes in response to the 2 triplet sequences.

The difference in total spike count between the responses to the triplets with the B tone 6 and 1 semitones above A is therefore 3.49 spikes, out of a total response of 43.78 spikes when B is not detectably different from A in frequency. Thus, there is an 8.0% decrease (3.49/43.78) in total spikes occurring associated with the switch from a perception of one tone stream to two separate streams of different tones. This is an overestimate of the change occurring in all of A1, because although a change of this magnitude will occur at the locations in (the tonotopically arranged) A1 where cells have best frequencies of A and B (Fishman et al., 2001), at other locations the difference in firing rate will be smaller.

### The relationship between cortical pyramidal cell firing rate and energy use

In the Results we use the mean changes of firing of cortical pyramidal cells calculated above to estimate the change of energy use occurring in a cortical area as a result of altered activity in the recorded cells themselves, and in their input synapses and surrounding glia. Cortical energy use initiated by action potentials has components proportional to postsynaptic and presynaptic action potential frequency (Attwell and Laughlin, 2001) (the relative magnitudes of which differ for rodent and primate cortex (Attwell and Laughlin, 2001), owing to the different cell and synapse densities present). The energy used to reverse the ion movements generating postsynaptic action potentials is proportional to postsynaptic firing rate, while energy use on presynaptic action potentials and transmitter release, transmitter and vesicle recycling, and reversal of postsynaptic ion movements generated by transmitter release, is proportional to presynaptic firing rate (assuming no change of vesicle release probability or postsynaptic current size over the small range of presynaptic firing rates considered) (Attwell and Laughlin, 2001).

The fractional change in the total (pre- and postsynaptic) action potential driven energy use will be equal to the fractional change in postsynaptic firing frequency, provided that fractional changes in postsynaptic frequency are equal to fractional changes in presynaptic frequency, i.e. excitatory synapses onto the recorded pyramidal cells generate an output frequency proportional to input frequency. Simulations of cortical neurons (Salinas and Sejnowski, 2000) and dynamic clamp experiments on cortical (Desai and Walcott, 2006) and cerebellar neurons (Mitchell and Silver, 2003) suggest that over much of the middle of the firing range of neurons this is a reasonable approximation, but that near the threshold for firing the fractional increase in postsynaptic rate is larger than the fractional increase in presynaptic rate (which would lead to our energy use values being overestimates, because we would overestimate the energy use driven by presynaptic action potentials) while the reverse is true at high firing rates where the synapses saturate (leading to our energy use values being underestimates). In addition, a “push–pull” organization of synaptic inputs (Andersen et al., 2000), where excitation increases while inhibition decreases, would result in postsynaptic firing increasing by a larger fraction than presynaptic firing, and so would also result in us overestimating the fractional energy increase occurring (simplistically, by a factor of ~ 2 if the increase of firing is produced half by the decrease of inhibition and half by the increase of excitation, assuming that reversing synaptic Na^+^ entry is the dominant energy consuming process in primate neurons (Attwell and Laughlin, 2001)). Detailed investigations have not been reported of the input–output relations for the synapses projecting to the pyramidal cells involved in the three perceptual tasks considered, however the following summary of the response properties of these cells establishes that during these tasks they are firing in the middle of their response range, far from the threshold for firing and far from saturation (which is more important for our conclusion that conscious perception is associated with little energy use since approaching saturation would, as outlined above, lead to us underestimating energy use). This implies, therefore, that the linear synapse assumption is reasonable. Consequently, if the recorded cells fire simply as a result of increased excitation, our energy estimates should be fairly accurate, while if a push–pull input arrangement occurs then the change in energy use associated with perception will be even smaller than we estimate.

#### Visual motion task

For the 14% coherence stimuli considered, which produce firing between 18 and 25 Hz, MT neurons are in the middle of their firing range: their spontaneous rate in darkness is 8 Hz, a 0% coherence stimulus results in firing at 20 Hz (see above), while a 100% coherence stimulus will (given the measured dependence of firing rate on coherence (Britten et al., 1993)) result in cells responsive to motion in that direction increasing their firing to ~ 60 Hz. Consequently fractional changes in postsynaptic firing frequency are likely to be approximately proportional to fractional changes in presynaptic frequency.

#### Skin vibration task

In the Results we consider flutter frequency discrimination around a frequency of 20 Hz. This frequency of vibration evokes a firing rate in area S2 pyramidal cells which is roughly in the middle of the firing rates evoked by low and high flutter frequencies, both for cells that increase their firing at higher vibration frequencies and for cells that decrease their firing at higher frequencies (Fig. 2B, E of Romo et al. (2003)). Since the input synapses are operating far from the threshold for evoking spikes and far from saturation, fractional changes in postsynaptic firing frequency are likely to be approximately proportional to fractional changes in presynaptic frequency.

#### Tone segregation task

The baseline firing rate of area A1 pyramidal neurons is about 25 Hz (Fig. 2 of Micheyl et al. (2005)). The difference in spike counts that generate the perceptual response to the B tone in A-B-A triplets occurs around a firing rate (averaged over the response to the B tone) of 60 Hz (Fig. 2 of Micheyl et al. (2005)), which is much lower than the cells' maximum firing rate of at least 150 Hz (the highest rate evoked by the A tone (Fig. 2 of Micheyl et al. (2005)). Thus, since the input synapses are operating far from the threshold for evoking spikes and far from saturation, fractional changes in postsynaptic firing frequency are likely to be approximately proportional to fractional changes in presynaptic frequency.

#### Electrophysiological sampling bias

The changes of firing rate that we use to estimate cortical energy consumption are probably dominated by recordings from neurons which are large (pyramidal cells) and which fire at a high rate. Omission of smaller cortical interneurons (smooth and spiny stellate cells) should not alter our conclusions significantly because they comprise only 25% of the neurons present (Abeles, 1991), and because their percentage change of firing rate may be similar to that of the principal neurons. The omission of cells that fire rarely or not at all will imply, provided that they increase their firing rate by less than the pyramidal cells, that the change of mean firing rate per neuron is lower than we assume, and lead to our deduced energy usage being an overestimate, reinforcing further the point that perception is associated with only a small change of cortical energy use.

### Stimuli that are well above the threshold for perception

For the detection of visual motion calculation described above, if the coherence is twice the threshold value in Eq. (1), i.e. 28% then, for a steepness parameter of *α* = 1.4 (from the neuron data in Fig. 9B of Britten et al. (1992), weighted by number of cells), Eq. (1) predicts that stimulus motion will be correctly detected 96.4% of the time (Fig. 2d). Repeating the calculation above for 28% coherence predicts that this highly reliable detection of the motion percept is associated with a 14% increase in mean firing rate.

Similarly we repeated the calculation for flutter perception given above, but for comparison of a 30 Hz vibration with a 20 Hz vibration, for which the correct behavioural choice (Fig. 2e) is made 97% of the time (from Fig. 4F of Romo et al. (2003)). Averaged over all the cells in S2, the mean increase of action potentials/neuron is then 0.56, for the 3-s period during which the two stimuli are applied, i.e. a 1.2% increase over the basal firing rate.

For the tone sequence experiment with tone B 6 semitones above A, psychophysical experiments on humans show that perception of the two tone streams reaches its maximum (94% correct) after 5 s of repeating the tone triplets (Fig. 2f, from Fig. 1 of Micheyl et al. (2005)). Extending to 5 s the counting of the spikes of monkey A1 neurons (from Fig. 3 of Micheyl et al. (2005)) showed that (including the 25 Hz baseline firing rate) the total spike count with B 6 semitones above A was 16.7 spikes less than the 195.8 spikes occurring when B was just one semitone above A, i.e. an 8.5% difference.

Thus, in all three cortical areas, the mean change in firing rate used for conscious perception of stimuli is low, not just at the threshold of perception, but also when perception is highly reliable.

## Results

### Visual task

For the visual modality, we analysed detection of movement in random dot visual stimuli, which has been studied psychophysically and electrophysiologically at the single unit level in monkeys (Britten et al., 1992, Britten et al., 1993), as well as psychophysically (Britten et al., 1992) and by fMRI (Rees et al., 2000) in humans. An array of moving dot stimuli is presented (Fig. 2a), with the dot intensity well above the threshold for visibility, in which a certain percentage (X) of dots move in the same direction (termed X% coherence), and the observer has to decide what the general direction of motion is. A neural substrate contributing to this decision is likely to be the middle temporal visual area (MT or V5). This is the first area on the visual pathway in which the responses of most monkey neurons (up to 97%) are directionally selective (Van Essen et al., 1981), and is activated when humans view moving stimuli (Zeki et al., 1991; Ptito et al., 2001). The sensitivity of monkey MT neurons to such stimuli correlates well with the monkeys' psychophysically determined threshold and the psychometric function relating performance to strength (coherence) of the motion signal (Britten et al., 1992) (Fig. 2d, data from Fig. 4 of Britten et al. (1992)). Furthermore, the psychophysical performance of human observers on this task is similar to that of monkeys (Britten et al., 1992), supporting extrapolation from the monkey neuronal firing data to the human cortex. From area MT, information is passed to the prefrontal cortex or lateral intraparietal area, and a behavioural decision based on the perceived motion is made (Kim and Shadlen, 1999, Huk and Shadlen, 2005) (Fig. 1).

**Fig. 3:**
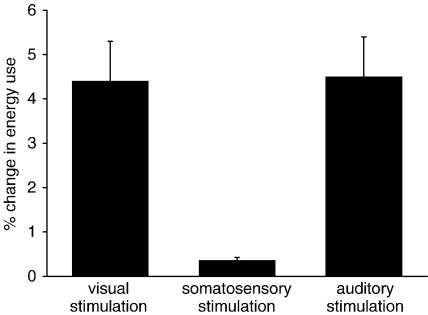
Estimated percentage changes in brain energy use associated with conscious perception of stimuli. Bars show mean increase in energy use associated with perception of a moving stimulus, increase in energy use associated with detection of a difference in flutter frequency, or decrease in energy use associated with detection of tone streaming (SEM was calculated from the 2 results obtained assuming that either 50 or 75% of total energy is action potential driven).

To calculate the energy needed to support the perception of motion in area MT, we need to know the action potential rate of the cells analysing the motion. Increasing the stimulus coherence increases the firing of cells tuned to respond to the direction of motion of the coherently moving dots, decreases the firing of cells tuned to the opposite direction (Fig. 2g), and makes it easier to detect the general direction of motion of the dots. Averaging over cells responding to all directions of motion, for a coherence value which is at the threshold of detection of the motion (i.e. for an 82% success rate in determining the direction of motion, see Materials and methods) we estimate that the motion percept is associated with a 7% increase in mean firing rate in area MT, compared to that occurring during presentation of a 0% coherence stimulus (for details of the calculation see Materials and methods). This implies, with the assumption that firing-related energy consumption is proportional to firing rate, as explained in Materials and methods, a 7% increase in firing-related energy use. If action potential driven signalling energy accounts for ~ 50–75% of the total energy consumption (Attwell and Laughlin, 2001), then this 7% increase of firing rate implies an increase of total local energy consumption of 50–75% of 7% = 3.5–5.3% (Fig. 3).

### Somatosensory task

For the somatosensory modality, we analysed the detection of “flutter”, a sensation which is produced when 5–50 Hz vibrations are applied to the skin to activate Meissner's mechanoreceptors (Mountcastle et al., 1967). In monkeys, information from these receptors is passed to somatosensory cortical area S1, where neurons are active during the period of vibration, and also to higher areas (Romo et al., 2004) (S2, prefrontal cortex, and ventral and medial premotor cortex: Fig. 1). In these higher areas the neural activity evoked outlasts the period of stimulation, providing a working memory trace that can be used to compare the frequency of a second stimulus (Fig. 2b) with that of an earlier stimulus (with both of the individual stimuli being well above the threshold for detection) (Romo et al., 2004). In humans, flutter vibration also activates (Maldjian et al., 1999) areas S1 and S2, and in psychophysical experiments (Fig. 2e, data replotted from Fig. 1e, Romo et al. (2002)) monkeys and humans distinguish the frequency of sequentially applied vibrations with a similar performance (Lamotte and Mountcastle, 1975). We estimated the energy expended in area S2 to generate neural activity which can support the discrimination of the frequency of two stimuli, i.e. the increase in energy expenditure associated with perception of the frequency difference, above that needed merely to represent two stimuli which are not perceived as different.

Neurons in monkey S2 change their firing when a flutter stimulus is applied, but only 40% of these cells show a response that depends on the vibration frequency (Romo et al., 2002) (of which 57% show an increase in firing rate, and 43% show a decrease in rate, with increasing frequency (Romo et al., 2003)). When two stimuli of different frequency, *f*1 and *f*2, are applied for 0.5 s, separated by a few seconds in time (Fig. 2b), the action potential firing rate in response to the second stimulus evolves over time to depend largely on the difference in frequency (this dependence is plotted as the parameter a2 in Fig. 2h, see Materials and methods). For an initial frequency of *f*1 = 20 Hz, we evaluated the mean difference in spike count averaged over all cells when *f*2 was sufficiently above *f*1 to be distinguished perceptually from a second presentation of a stimulus with *f*2 = *f*1 = 20 Hz. The calculation shows that there is a 0.57% increase in mean firing rate at the threshold of perception in area S2 (see Materials and methods for full calculation details). As above for visual perception, if action potential driven energy use is ~ 50–75% of the total energy consumption (Attwell and Laughlin, 2001), the 0.57% increase of firing occurring during flutter detection implies an increase of local energy consumption of 50–75% of 0.57% = 0.29–0.43% (Fig. 3).

### Auditory task

For the auditory modality we studied tone stream segregation. A repeated sequence of (suprathreshold) sound tones alternating between two frequencies, A and B, such as ABA–ABA–ABA– (Fig. 2c), is heard by humans as two separate streams of constant pitch tones if A and B are sufficiently different (i.e. heard as A–A–A– and B–B–B–, with the stream separation building up over time, Fig. 2f, data from Micheyl et al. (2005) kindly provided by C. Micheyl), but as a single tone stream with a galloping rhythm if A and B are close (Bregman and Campbell, 1971). Tone stream segregation has also been demonstrated in psychophysical experiments on monkeys (Izumi, 2002). We estimated the change in energy expenditure associated with tone stream segregation in the primary auditory cortex (A1) using electrophysiological data from monkeys (Fig. 2i) (Micheyl et al., 2005) recorded during exposure to tone triplets with B tones either 6 semitones or 1 semitone above the frequency of the A tone, for which tone stream segregation does, or does not occur, respectively (Micheyl et al., 2005). These data revealed that there is an 8.0% decrease in total spikes occurring in A1 associated with the switch from a perception of one tone stream to perceiving two separate streams of different tones (see Materials and methods). As above for visual perception, if action potential driven energy use is ~ 50–75% of the total energy consumption (Attwell and Laughlin, 2001), auditory stream detection will lead to a decrease of local energy consumption of 50–75% of 8% = 4–6% (Fig. 3).

### Supra-threshold stimuli

The calculations above detail the change in cortical energy use associated with the threshold perception of a stimulus attribute. Larger energy changes are expected for stimuli above threshold, but repeating the calculations for this case shows that even well above threshold, where stimulus detection is highly reliable, the fractional change of energy use is still surprisingly small: 7–10.6% for the visual motion task, 0.6–0.9% for the flutter perception task, and 4.3–6.4% for the tone stream segregation task (calculated as above from the mean firing rate changes given in Materials and methods).

## Discussion

### The energy used on conscious perception

We have estimated how much brain energy is needed to produce information which can be used to generate a percept, over and above the energy needed to represent the incoming stimuli when the percept is absent, for example the energy needed for visual processing of dot stimuli which move sufficiently coherently to generate a percept of general movement, relative to that needed for processing of the same number of dots which move randomly and so generate no general movement percept. All three cortical areas studied show only a small change of energy usage (Fig. 3) associated with perception (< 6% at threshold, and < 11% when perception is reliable), despite the difference in modalities that they serve (vision, touch, audition) and large differences in baseline firing rate, suggesting that this result is of general relevance.

A major reason for the small size of this energy change, at least for the visual motion and somatosensory flutter stimuli considered, is that sensory attributes are encoded across a population of neurons as a mixture of increased and decreased firing. Across the population of neurons considered, therefore, the changes in mean firing rate and energy usage are much less than would occur if firing simply increased in all neurons with the strength of the sensory attribute. Thus, encoding stimuli across a population of neurons as a mixture of increased and decreased firing confers the advantage that the energy demand of the brain is kept more constant and requires smaller blood flow changes (rather than requiring that blood flow increases dramatically when a stimulus is given), in addition to aiding the removal of noise that is common to all cells (Romo et al., 2003) and ensuring high temporal resolution at all stimulus strengths (Attwell, 1986).

For these conclusions to hold for humans, we must assume that experimental measurements of the change of action potential frequency evoked by sensory stimuli in awake monkeys can be extrapolated to humans. While there is no easy way to verify this, the similar psychophysical results (Britten et al., 1992; Lamotte and Mountcastle, 1975; Bregman and Campbell, 1971; Izumi, 2002) obtained in monkey and human for all three sensory stimuli studied suggest that the cellular mechanisms underlying perception are similar in the two species. The other important assumption that we make (based on the analysis in Materials and methods of the neural firing rates in the tasks considered) is that the fractional change of firing rate of the principal cells recorded from is proportional to the fractional change of firing rate in the presynaptic cells sending input to these cells. This assumption (which requires future experimental work to test it) results in the fractional change in neuronal energy use being proportional to the fractional change of firing rate of the principal neurons. It also means that the total fractional change of energy use calculated is independent of the exact proportions of energy expended on presynaptic processes, postsynaptic currents or action potentials.

If the change in energy use is so small, why has evolution produced systems that increase blood flow when neurons are active? It is important to note that our predictions of a small increase in firing rate and energy expenditure are only for the perception of a feature of the incoming sensory information. Thus, in the vision example given above, the presence of a zero coherence array of randomly moving dots increases the mean firing rate in area V5 from ~ 8 to ~ 20 Hz (Huk and Shadlen, 2005). Similarly, neurons representing tactile flutter stimuli can double their firing frequency when the stimulus is present (Fig. 7b of Romo et al. (2002)), and A1 neurons increase their firing 6-fold when a tone is applied (Fig. 2 of Micheyl et al. (2005)). Converting these firing rate changes to energy use changes is complicated by the fact that, unlike for the changes associated with perception discussed above, these firing rates may not be exclusively in the range where the input–output relationship of the synapses is linear (see Materials and methods). Nevertheless the much larger firing rate changes associated with unconscious information processing than with perception suggest that the changes in energy expenditure associated with conscious perception are much smaller than those associated with the unconscious representation of incoming sensory information, for which the increase in blood flow associated with neuronal activity will be more important.

### Relevance to BOLD fMRI

Functional imaging experiments often assess the difference in brain “activation” for two situations designed to differ only in the perception of some stimulus attribute (Tootell et al., 1995; Phillips et al., 1997; Epstein and Kanwisher, 1998, McKeefry and Zeki, 1997). Our calculations show that, in the tasks we consider, perception is associated with only a small change in energy usage (the exact size of which varies between cortical areas, being an order of magnitude smaller for the somatosensory flutter task than for the visual motion task, and even a decrease in the case of tone stream segregation). For a small increase of neural firing and energy usage it is unclear whether an increase in blood supply is actually needed to power the neural activity, or whether an increased extraction of oxygen and glucose from the blood (driven by the lowered local concentration of these substrates) would suffice. It does, however, seem likely that any neurotransmitter-mediated (Drake and Iadecola, 2007) increase of blood flow associated with perception need only be small. Interestingly, the blood flow increases generating functional imaging signals are rather small (Raichle and Mintun, 2006), typically less than 5–10%, and the BOLD signal increase associated with the perception at threshold of the stimulus properties considered here is less than 1% in the visual motion (Rees et al., 2000), flutter discrimination (Hegner et al., 2007) and tone stream segregation (Wilson et al., 2007) tasks. It seems possible, therefore, that perception of a stimulus attribute in some brain areas, particularly those where a decrease in the firing of some neurons outweighs an increase in the firing of other neurons and so is likely to lead to a decrease in the total release of glutamate (which is the main agent thought to trigger vasodilator release and BOLD signals: see Introduction), may fail to be detected as a positive BOLD fMRI signal (cf. Shmuel et al., 2006).

## References

[bib1] Abeles M. (1991). Corticonics: Neural Circuits of the Cerebral Cortex.

[bib2] Albright T.D., Desimone R., Gross C.G. (1984). Columnar organization of directionally selective cells in visual area MT of the macaque. J. Neurophysiol..

[bib3] Andersen J.S., Carandini M., Ferster D. (2000). Orientation tuning of input conductance, excitation, and inhibition in cat primary visual cortex. J. Neurophysiol..

[bib4] Arieli A., Sterkin A., Grinvald A., Aertsen A. (1996). Dynamics of ongoing activity: explanation of the large variability in evoked cortical responses. Science.

[bib5] Attwell D. (1986). The Sharpey-Schafer lecture. Ion channels and signal processing in the outer retina. Q. J. Exp. Physiol..

[bib6] Attwell D., Laughlin S.B. (2001). An energy budget for signaling in the grey matter of the brain. J. Cereb. Blood Flow Metab..

[bib7] Barlow H.B. (1957). Increment thresholds at low intensities considered as signal/noise discriminations. J. Physiol..

[bib8] Bregman A.S., Campbell J. (1971). Primary auditory stream segregation and perception of order in rapid sequences of tones. J. Exp. Psychol..

[bib9] Britten K.H., Newsome W.T. (1998). Tuning bandwidths for near-threshold stimuli in area MT. J. Neurophysiol..

[bib10] Britten K.H., Shadlen M.N., Newsome W.T., Movshon J.A. (1992). The analysis of visual motion: a comparison of neuronal and psychophysical performance. J. Neurosci..

[bib11] Britten K.H., Shadlen M.N., Newsome W.T., Movshon J.A. (1993). Responses of neurons in macaque MT to stochastic motion signals. Vis. Neurosci..

[bib12] Cusack R. (2005). The intraparietal sulcus and perceptual organization. J. Cogn. Neurosci..

[bib13] Desai N.S., Walcott E.C. (2006). Synaptic bombardment modulates muscarinic effects in forelimb motor cortex. J. Neurosci..

[bib14] Drake C.T., Iadecola C. (2007). The role of neuronal signaling in controlling cerebral blood flow. Brain Lang..

[bib15] Epstein R., Kanwisher N. (1998). A cortical representation of the local visual environment. Nature.

[bib16] Faraci F.M., Breese K.R. (1993). Nitric oxide mediates vasodilatation in response to activation of N-methyl-D-aspartate receptors in brain. Circ. Res..

[bib17] Fishman Y.I., Reser D.H., Arezzo J.C., Steinschneider M. (2001). Neural correlates of auditory stream segregation in primary auditory cortex of the awake monkey. Hear Res..

[bib18] Fiser J., Chiu C., Weliky M. (2004). Small modulation of ongoing cortical dynamics by sensory input during natural vision. Nature.

[bib19] Fox P.T., Raichle M.E., Mintun M.A., Dence C. (1988). Nonoxidative glucose consumption during focal physiologic neural activity. Science.

[bib20] Gjedde A., Marrett S., Vafaee M. (2002). Oxidative and nonoxidative metabolism of excited neurons and astrocytes. J. Cereb. Blood Flow Metab..

[bib21] Gutschalk A., Micheyl C., Melcher J.R., Rupp A., Scherg M., Oxenham A.J. (2005). Neuromagnetic correlates of streaming in human auditory cortex. J. Neurosci..

[bib22] Hegner Y.L., Saur R., Veit R., Butts R., Leiberg S., Grodd W., Braun C. (2007). BOLD adaptation in vibrotactile stimulation: neuronal networks involved in frequency discrimination. J. Neurophysiol..

[bib23] Hubel D.H., Wiesel T.N. (1998). Early exploration of the visual cortex. Neuron.

[bib24] Huk A.C., Shadlen M.N. (2005). Neural activity in macaque parietal cortex reflects temporal integration of visual motion signals during perceptual decision making. J. Neurosci..

[bib25] Izumi A. (2002). Auditory stream segregation in Japanese monkeys. Cognition.

[bib26] Kasischke K.A., Vishwasrao H.D., Fisher P.J., Zipfel W.R., Webb W.W. (2004). Neural activity triggers neuronal oxidative metabolism followed by astrocytic glycolysis. Science.

[bib27] Kim J.N., Shadlen M.N. (1999). Neural correlates of a decision in the dorsolateral prefrontal cortex of the macaque. Nat. Neurosci..

[bib28] Lamotte R.H., Mountcastle V.B. (1975). Capacities of humans and monkeys to discriminate vibratory stimuli of different frequency and amplitude: a correlation between neural events and psychological measurements. J. Neurophysiol..

[bib29] Maldjian J.A., Gottschalk A., Patel R.S., Pincus D., Detre J.A., Alsop D.C. (1999). Mapping of secondary somatosensory cortex activation induced by vibrational stimulation: an fMRI study. Brain Res..

[bib30] Malonek D., Grinvald A. (1996). Interactions between electrical activity and cortical microcirculation revealed by imaging spectroscopy: implications for functional brain mapping. Science.

[bib31] McKeefry D.J., Zeki S. (1997). The position and topography of the human colour centre as revealed by functional magnetic resonance imaging. Brain.

[bib32] Micheyl C., Tian B., Carlyon R.P., Rauschecker J.P. (2005). Perceptual organization of tone sequences in the auditory cortex of awake macaques. Neuron.

[bib33] Mitchell S.J., Silver R.A. (2003). Shunting inhibition modulates neuronal gain during synaptic excitation. Neuron.

[bib34] Mountcastle V.B., Talbot W.H., Darian-Smith I., Kornhuber H.H. (1967). Neural basis of the sense of flutter-vibration. Science.

[bib35] Phillips M.L., Young A.W., Senior C., Brammer M., Andrew C., Calder A.J., Bullmore E.T., Perrett D.I., Rowland D., Williams S.C., Gray J.A., David A.S. (1997). A specific neural substrate for perceiving facial expressions of disgust. Nature.

[bib36] Ptito M., Kupers R., Faubert J., Gjedde A. (2001). Cortical representation of inward and outward radial motion in man. Neuroimage.

[bib37] Raichle M.E., Mintun M.A. (2006). Brain work and brain imaging. Annu. Rev. Neurosci..

[bib38] Rees G., Friston K., Koch C. (2000). A direct quantitative relationship between the functional properties of human and macaque V5. Nat. Neurosci..

[bib39] Romo R., Hernandez A., Zainos A. (2004). Neuronal correlates of a perceptual decision in ventral premotor cortex. Neuron.

[bib40] Romo R., Hernandez A., Zainos A., Lemus L., Brody C.D. (2002). Neuronal correlates of decision-making in secondary somatosensory cortex. Nat. Neurosci..

[bib41] Romo R., Hernandez A., Zainos A., Salinas E. (2003). Correlated neuronal discharges that increase coding efficiency during perceptual discrimination. Neuron.

[bib42] Salinas E., Sejnowski T. (2000). Impact of correlated synaptic input on output firing rate and variability in simple neuronal models. J. Neurosci..

[bib43] Shmuel A., Augath M., Oeltermann A., Logothetis N.K. (2006). Negative functional MRI response correlates with decreases in neuronal activity in monkey visual area V1. Nature Neurosci..

[bib44] Tootell R.B., Reppas J.B., Dale A.M., Look R.B., Sereno M.I., Malach R., Brady T.J., Rosen B.R. (1995). Visual motion aftereffect in human cortical area MT revealed by functional magnetic resonance imaging. Nature.

[bib45] Van Essen D., Maunsell J.H.R., Bixby J.L. (1981). The middle temporal visual area in the macaque: myeloarchitecture, connections, functional properties and topographic organization. J. Comp. Neurol..

[bib46] Wilson E.C., Melcher J.R., Micheyl C., Gutschalk A., Oxenham A.J. (2007). Cortical fMRI activation to sequences of tones alternating in frequency: Relationship to perceived rate and streaming. J. Neurophysiol..

[bib47] Zeki S.M., Watson J.D.G., Lueck C.J., Friston K.J., Kennard C., Frackowiak R.S.J. (1991). A direct demonstration of functional specialization in human visual cortex. J. Neurosci..

[bib48] Zonta M., Angulo M.C., Gobbo S., Rosengarten B., Hossmann K.A., Pozzan T., Carmignoto G. (2003). Neuron-to-astrocyte signaling is central to the dynamic control of brain microcirculation. Nat. Neurosci..

